# Supplementary material for: Spatiotemporal analysis for detection of pre-symptomatic shape changes in neurodegenerative diseases: Initial application to the GENFI cohort
Source: Neuroimage. 2019 Mar;188:282–90. doi: 10.1016/j.neuroimage.2018.11.063 (PMC6414401; doi:10.1016/j.neuroimage.2018.11.063)

Supplementary materials:

Supplementary materials of *"Spatiotemporal analysis for detection of pre-symptomatic shape changes in neurodegenerative diseases: initial application to the GENFI cohort"* paper.

These tables show complementary results for different number of cluster, following the spation-tempral analysis applied to the thalamus in genetic fronto-temporal dementia, presented in the "Spatiotemporal analysis for detection of pre-symptomatic shape changes in neurodegenerative diseases: applied to GENFI study" study. The table presenting results for 10 clusters is in the main paper as it is part of the main study. Tables 4, 5 and 6 show significant differences over time in the orientation of clusters displayed at Figure 1. All 3 significant clusters have differences in the frontal part of the thalamus as Cluster 1 presented in the paper, and those differences are shown 5 years before expect onset.

			C_1^2	C_2^2
2 clusters	Am	T1	.	.
		T2	.	.
	Or	T1	.	.
		T2	.	.

Table 1: Using 2 clusters to group the spatio-temporal deformation, T1: Wald test for differences between MC and controls and T2: for differences over time between MC and controls. None of them show significant differences between mutation carriers and controls.

			C_1^4	C_2^4	C_3^4	C_4^4
4 clusters	Am	T1	.	.	.	0.003
		T2	.	.	.	0.002
	Or	T1	.	.	.	.
		T2	.	.	.	.

Table 2: Using 4 clusters to group the spatio-temporal deformation, T1: Wald test for differences between MC and controls and T2: for differences over time between MC and controls. One of the custer shows significant differences in amplitude between mutation carriers and controls, but after correction for multiple comparisons, the significance is lost.

			C_1^6	C_2^6	C_3^6	C_4^6	C_5^6	C_6^6
6 clusters	Am	T1	.	.	0.01	.	.	.
		T2	.	.	0.004	.	.	.
	Or	T1	0.04	0.03	.	.	.	.
		T2	.	0.007	.	.	0.03	.

Table 3: Using 6 clusters to group the spatio-temporal deformation, T1: Wald test for differences between MC and controls and T2: for differences over time between MC and controls. Several custers show significant differences without correction. The significance is lost avec correction for multiple comparison.

			C_1^8	C_2^8	C_3^8	C_4^8	C_5^8	C_6^8	C_7^8	C_8^8
8 clusters	Am	T1	.	.	.	.	.	.	.	.
		T2	.	.	.	.	.	.	.	.
	Or	T1	.	.	**≤1e-5**	.	.	.	.	.
		T2	.	.	**≤1e-5**	.	.	.	.	.

Table 4: Using 8 clusters to group the spatio-temporal deformation, T1: Wald test for differences between MC and controls and T2: for differences over time between MC and controls. One of the custer shows significant differences in orientation between mutation carriers and controls after correction for multiple comparisons The deformation corresponding to the cluster is shown Figure 1.

			C_1^{12}	C_2^{12}	C_3^{12}	C_4^{12}	C_5^{12}	C_6^{12}	C_7^{12}	C_8^{12}	C_9^{12}	C_{10}^{12}	C_{11}^{12}	C_{12}^{12}
12 clusters	Am	T1	.	.	.	.	.	.	.	.	.	.	.	.
		T2	.	.	.	.	.	.	.	.	.	.	0.05	.
	Or	T1	.	.	.	**≤1e-5**	.	.	.	.	.	.	.	.
		T2	.	.	.	**1e-4**	.	.	.	.	.	.	.	.

Table 5: Using 12 clusters to group the spatio-temporal deformation, T1: Wald test for differences between MC and controls and T2: for differences over time between MC and controls. One of the custer shows significant differences in orientation between mutation carriers and controls after correction for multiple comparisons The deformation corresponding to the cluster is shown Figure 1.

			C_1^{14}	C_2^{14}	C_3^{14}	C_4^{14}	C_5^{14}	C_6^{14}	C_7^{14}	C_8^{14}	C_9^{14}	C_{10}^{14}	C_{11}^{14}	C_{12}^{14}	C_{13}^{14}	C_{14}^{14}
14 clusters	Am	T1	.	.	.	.	.	.	.	.	.	.	.	.	.	.
		T2	.	.	.	.	.	.	.	.	.	.	.	.	.	.
	Or	T1	.	.	.	.	.	.	.	.	**≤1e-5**	.	0.01	.	.	.
		T2	.	.	.	.	.	.	.	.	**≤1e-5**	.	0.004	.	.	.

Table 6: Using 14 clusters to group the spatio-temporal deformation, T1: Wald test for differences between MC and controls and T2: for differences over time between MC and controls. One of the custer shows significant differences in orientation between mutation carriers and controls after correction for multiple comparisons The deformation corresponding to the cluster is shown Figure 1.

			C_1^{16}	C_2^{16}	C_3^{16}	C_4^{16}	C_5^{16}	C_6^{16}	C_7^{16}	C_8^{16}	C_9^{16}	C_{10}^{16}	C_{11}^{16}	C_{12}^{16}	C_{13}^{16}	C_{14}^{16}	C_{15}^{16}	C_{16}^{16}
16 clusters	Am	T1	.	.	.	.	.	.	.	.	.	.	.	.	0.01	.	.	.
		T2	.	.	.	.	.	.	.	.	.	.	.	.	0.009	.	.	.
	Or	T1	.	.	.	.	.	.	.	.	.	.	.	.	.	0.005	.	.
		T2	.	.	.	.	.	.	.	.	.	.	.	.	.	0.008	.	.

Table 7: Using 16 clusters to group the spatio-temporal deformation, T1: Wald test for differences between MC and controls and T2: for differences over time between MC and controls. Two clusters shows significant differences which p-values are not small enough to survive multiple comparison correction.

Figure 1: Thalamic connectivity atlas, and deformations of clusters $C_3^8 C_4^{12}$ and C_9^{14} from the above Tables. The color map shows displacement in mm of the deformation. The same scale is used for the 3 clusters. Graphes shows Wald test results for different EYO.

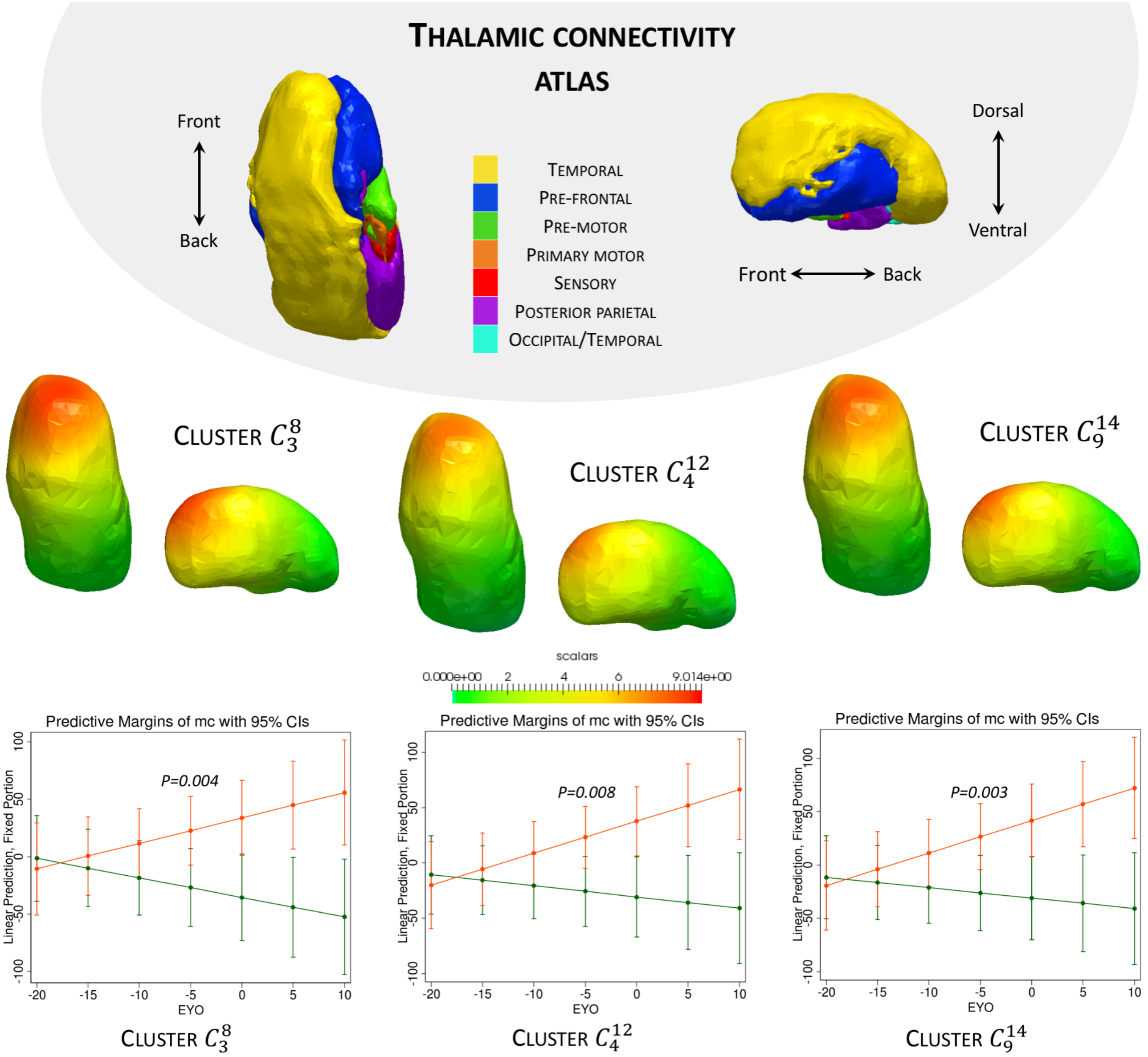

Supplement: Online data [file mmc1.pdf]
